# Spurious autobiographical memories of psychosis: a dopamine-gated neuroplasticity account for relapse and treatment-resistant psychosis

**DOI:** 10.1017/S0033291724003027

**Published:** 2025-04-07

**Authors:** Yu Hai Eric Chen, Stephanie M.Y. Wong, Melody M. So, Yi Nam Suen, Christy L.M. Hui

**Affiliations:** 1 Centre for Youth Mental Health, University of Melbourne, Parkville, VIC 3052, Australia; 2 Orygen, Parkville, VIC 3052, Australia, 3 School of Clinical Medicine HKU; 3 School of Clinical Medicine, LKS Faculty of Medicine, The University of Hong Kong, Hong Kong SAR; 4 Department of Social Work and Social Administration, The University of Hong Kong, Hong Kong SAR; 5 School of Nursing, LKS Faculty of Medicine, The University of Hong Kong, Hong Kong SAR

**Keywords:** autobiographical memory, dopamine, relapse, treatment-resistant psychosis

## Abstract

Psychotic disorders are known to be associated with elevated dopamine synthesis; yet, nondopamine factors may underlie the manifestation of some psychotic symptoms that are nonresponsive to dopamine-blocking agents. One under-explored nondopamine mechanism is neuroplasticity. We propose an account of the course of psychotic symptoms based on the extensive evidence for dopamine facilitation of Hebbian synaptic plasticity in cortical and subcortical memory systems. The encoding of psychotic experiences in autobiographical memory (AM) is expected to be facilitated in the hyperdopaminergic state associated with acute psychosis. However, once such ‘spurious AM of psychosis’ (SAMP) is encoded, its persistence may become dependent more on synaptic factors than dopamine factors. Under this framework, the involuntary retrieval of residual SAMP is postulated to play a key role in mediating the reactivation of symptoms with similar contents, as often observed in patients during relapse. In contrast, with active new learning of normalizing experiences across diverse real-life contexts, supported by intact dopamine-mediated salience, well-integrated SAMP may undergo ‘extinction’, leading to remission. The key steps to the integration of SAMP across psychotic and nonpsychotic memories may correspond to one’s ‘recovery style’, involving processes similar to the formation of ‘non-believed memory’ in nonclinical populations. The oversuppression of dopamine can compromise such processes. We synthesize this line of evidence into an updated dopamine-gated memory framework where neuroplasticity processes offer a parsimonious account for the recurrence, persistence, and progression of psychotic symptoms. This framework generates testable hypotheses relevant to clinical interventions.

## Background

A diverse range of longitudinal courses has been observed in schizophrenia and related psychotic disorders (Heilbronner, Samara, Leucht, Falkai, & Schulze, 2016). For positive symptoms, the course can vary between remission, persistent symptoms, and relapses with variable residual symptoms. An initial ‘full remission’ course may later evolve into a ‘residual symptom’ course, often after relapses (Emsley, Chiliza, & Asmal, 2013; Emsley, Chiliza, Asmal, & Harvey, 2013; Hui et al., 2018; Taipale, Tanskanen, Correll, & Tiihonen, 2022; Wiersma, Nienhuis, Slooff, & Giel, 1998). Compared with the many studies on factors leading to the onset of psychosis, relatively few have addressed illness courses after onset. A coherent account of psychosis should explain not only the onset of psychotic disorders but also the subsequent relapses and development of refractory symptoms, which can become increasingly independent of dopamine. The observation that symptoms during relapse often repeat contents similar to those in the previous episodes (Chaturvedi & Sinha, 1990; Grunfeld et al., 2024; Palaniyappan, 2019; Sinha & Chaturvedi, 1990) suggests that memory processes may be involved.

It is increasingly recognized that memory processes are modulated by dopamine (Sayegh et al., 2024; Shohamy & Adcock, 2010), which has already been observed to play a key role in producing psychotic symptoms (Howes, Bukala, & Beck, 2024; Howes & Kapur, 2009; Wong et al., 2022) through an excessive sense of salience (Howes & Nour, 2016; Kapur, 2003). We have previously argued broadly that known neuroplasticity processes interacting with dopamine may contribute to accounting for the course of psychotic symptoms (Chen et al., 2023). In the current narrative review, we elaborate on how a dopamine-gated autobiographical memory (AM) account coherently brings forth specific hypotheses relevant to the longitudinal evolution of psychotic symptoms.

### Memory gating roles of dopamine

#### Dopamine salience accounts

In everyday life, the brain monitors our environment by continuously predicting the next environmental state. The discrepancy between the predicted and the actual events generates a prediction error signal indicating the presence of novel information and preparing the brain for neuroplasticity (Heinz et al., 2019; Shohamy & Adcock, 2010). Such prediction error signals are associated with increased dopamine activities (Starkweather, Babayan, Uchida, & Gershman, 2017). When the dopamine system is active, correlated information in the environment is more readily encoded (Shohamy & Adcock, 2010). Notably, prediction error signals are supervened by a sense of salience: a subjective feeling that significant information has emerged in the environment (Howes et al., 2024; Howes & Kapur, 2009; Wong et al., 2022). Dopamine activation is also associated with increased functional connectivity in the salience network in the brain (Conio et al., 2020). During acute psychosis, the aberrant increase in dopamine activity therefore leads to a ‘spurious’ sense of salience and overinterpretation of neutral environmental information, an account that is consistent with narratives of people with psychotic disorders (Heinz et al., 2019; Howes et al., 2024; Kapur, 2003; McCutcheon, Krystal, & Howes, 2020).

While the prediction error-salience theory explains psychotic symptoms at illness onset, it does not fully explain how these symptoms evolve over time. Among the few accounts, the persistence of delusions has been explored using the prediction error model (Corlett, Krystal, Taylor, & Fletcher, 2009). The model posits that representations of psychotic and nonpsychotic experiences compete for dominance. Chronically, increased prediction error signals (presumably related to a sustained elevation in dopamine activity) are suggested to contribute to the persistence of delusions. However, the underlying assumption of a chronic state of dopamine overactivity remains contentious (Avram et al., 2019). The model also does not adequately account for the progressive increase in psychotic symptoms with each relapse.

### Memory-based accounts

Importantly, while new learning is facilitated by dopamine, once encoding has taken place, ongoing dopamine activity is not required to maintain the memory representation. This is in contrast to the persistent prediction error-salience models above. The subsequent course of the spurious memories formed during a psychotic episode can be understood with reference to the known natural history of memory traces in the brain.

Studies of memory in psychosis have largely focused on deficits based on failure to recall in standardized memory tests, rather than on aberrant memories (Danion,Huron, Vidailhet, & Berna, 2007; Harvey et al., 2022; Ranganath, Minzenberg, & Ragland, 2008). Previous studies addressing aberrant memory in psychosis were initiated with attempts to understand psychotic symptom formation using associative memory network models (Chen et al., 2009; Chen & Berrios, 1998; Hoffman et al., 1995; Hoffman & McGlashan, 1997; Rolls, 2021; Rolls, Loh, Deco, & Winterer, 2008). In line with this direction, it has been suggested that psychosis could be conceptualized as a ‘learning and memory disorder’ and could be understood in terms of known memory functions in the hippocampus (Tamminga, 2013). However, there has been little discussion on how this approach can be linked to the emerging dopamine-prediction error-salience findings. Independently, the role of traumatic experiences in psychotic disorders has increasingly been considered from a memory perspective (Hardy, 2017). Aberrant memory has also been incorporated into an account of brain state homeostasis fluctuating between neural overactivity and stabilization (Palaniyappan, 2019). In the context of these approaches, we seek to review whether known memory processes interacting with dopamine activity may be sufficient to offer a parsimonious account of the longitudinal course of psychosis symptoms that can bring forth new clinical insights and hypotheses. The core account is concisely outlined in the next section.

## A dopamine-gated memory account: Spurious Autobiographical Memories of Psychosis (SAMP)

**Figure 1.**
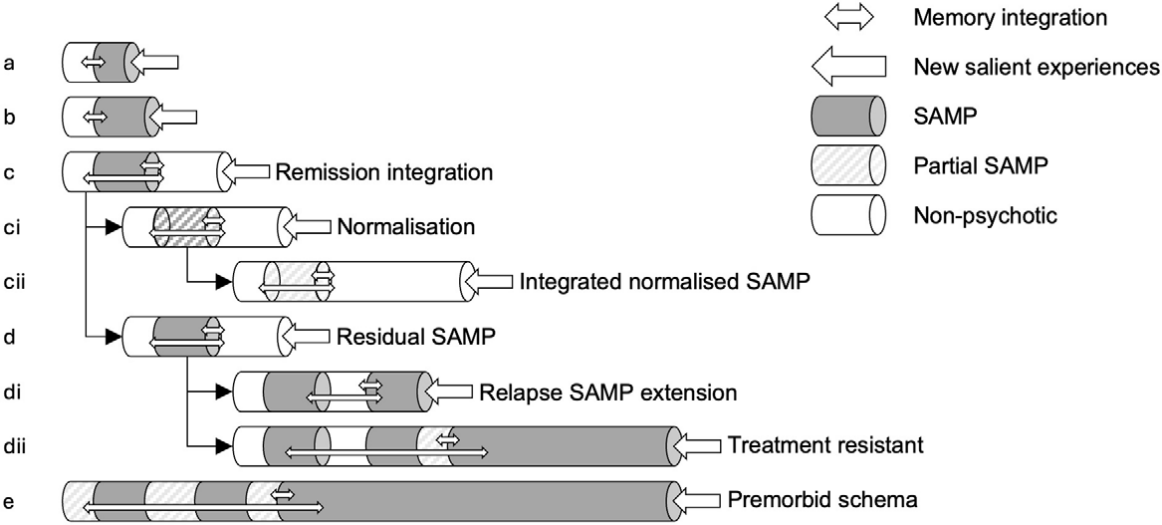
Illustration of Spurious Autobiographical Memory of Psychosis (SAMP). Dark backgrounds denote SAMP memories formed during psychosis. Each unit represents autobiographical memory (AM) encoded over a period of time. Arrows on the right of each row indicate current life experience. A light gray background indicates partially normalized AM. Arrows represent integrative processes between new and old memories. From the top, (a) SAMP associated with short duration of active psychosis (DAP); (b) SAMP associated with a longer DAP; (c) AM integration in remission: (ci) SAMP in remission; well-integrated SAMP enables extinction and reduction in SAMP (cii); (d) poorly integrated SAMP does not benefit effectively from normalization, instead, (di) relapses can add to SAMP; and (dii) increasing treatment resistance; (e) premorbid threat-prone schema generally facilitates SAMP reactivation and persistence.

We propose that during an acute psychotic episode, the contents of psychotic experiences and related aberrant associations are encoded as SAMP based on Hebbian synaptic plasticity, facilitated by elevated dopamine levels (Dringenberg, 2020; Duszkiewicz, McNamara, Takeuchi, & Genzel, 2019; Kamiński et al., 2018; Shohamy & Adcock, 2010; Wittmann et al., 2005). After remission, the newly encoded SAMP interacts with premorbid nonpsychotic AM to construct a coherent overall autobiographical account (Figure 1). Suboptimal integration results in a SAMP segregated from nonpsychotic AM. Access to SAMP during remission is also postulated to be impeded due to contextual mismatch. This limited access to SAMP in remission compromises the ability of normalized experience to modify the SAMP. Residual SAMP may become reactivated associatively and involuntarily upon encountering external or internal cues, leading to an elevated propensity for relapse (Chen et al., 2023).

### The formation of SAMP

#### Dopamine-gated neuroplasticity in SAMP

Recent accounts of everyday memory have emphasized the synergistic interaction between memory subsystems (Ferbinteanu, 2020; Rubin, 2006), which include memories of personal episodic events (Shohamy & Adcock, 2010), semantic memory (Battaglia & Pennartz, 2011), implicit associations (Ferbinteanu, 2020), and emotional memory (Luminet, 2022). Comparative brain anatomy suggested that although specialized memory systems have emerged in different phylogenetic stages to serve different adaptive functions, the basic function of dopamine gating of neuroplasticity has already appeared in early vertebrates. Interestingly, dopamine gating of memory appears to have been conserved during the evolution of specialized vertebrate memory systems, including the phylogenetically more recent human AM system (Allen & Fortin, 2013; Murray, Wise, & Graham, 2017).

Gated by dopamine, novel information is encoded in synapses through long-term potentiation (LTP) (Duszkiewicz et al., 2019; Kamiński et al., 2018; Sayegh et al., 2024; Sheynikhovich, Otani, & Arleo, 2013). LTP strengthens a specific synapse when its presynaptic signal is associated with a successful post-synaptic activation (the Hebbian rule) (Sayegh et al., 2024; Shohamy & Adcock, 2010). Dopamine facilitates LTP through postsynaptic mechanisms involving molecules such as the Calmodulin protein kinase II (CAMKII) and the cAMP response element-binding protein (CREB). Similar molecular pathways have been identified in the hippocampus (Prince, Bacon, Tigaret, & Mellor, 2016; Sayegh et al., 2024), the striatum (Speranza, Di Porzio, Viggiano, De Donato, & Volpicelli, 2021), and the amygdala (Allen & Fortin, 2013; Maren, 2015; Markowitsch & Staniloiu, 2011; Speranza et al., 2021). Thus, through LTP-mediated memory facilitation, dopamine interacts not only with implicit memory and emotional memory but also with the more recently evolved AM (Dringenberg, 2020; Hannula, Minor, & Slabbekoorn, 2023; Klein, Cosmides, Tooby, & Chance, 2002; Paré & Headley, 2023; Rubin, 2006).

Temporal integration between new and old AM, as well as between AM and implicit, emotional and semantic memories, may depend on synchronized neural oscillation (Fries, 2009; Fuentemilla, Palombo, & Levine, 2018; Steinvorth, Wang, Ulbert, Schomer, & Halgren, 2010). A reduced neural oscillation phase coherence is observed to be relatively specific for schizophrenia (Wolff & Northoff, 2024). This abnormality is postulated to be linked to a general temporal disorganization in a spatiotemporal psychopathology model proposed as a bridge between subjective and objective phenomena (Northoff, Daub, & Hirjak, 2023; Northoff & Hirjak, 2023). AM provides another portal to explore the interface between first-person phenomenological experience and mechanistic brain processes in psychotic disorders.

The focus on AM in the present account of psychosis is relevant not only because AM content is directly related to the conscious experience of psychotic symptoms but also because it contains identifiable time stamps that enable hypothesis testing by distinguishing between memories laid down in the psychotic and nonpsychotic periods (McWilliams et al., 2022).

### The structure of AM

AM involves personal memories of events organized into event groups and schemata (Brown, 2023; Conway, 2005; Rubin & Umanath, 2015; Zacks, 2020). Memories from different time periods are integrated with the self-schema to generate individual life narratives (Conway, 2005). AM thus contains information on event memories (Bird, 2020); and how these event memories are integrated through major transitions in life (Habermas, 2011; Habermas & Köber, 2015).

At the neurobiological level, event encoding is automatically segmented into epochs, each consisting of relatively continuous contextual information (Bird, 2020; Zacks, 2010, 2020). Events are demarcated by a shift in contextual information as flagged by increased prediction error signals (Kumar et al., 2023). Besides the hippocampus’ involvement in AM encoding, AM retrieval is mediated by the posterior cingulate gyrus and the ventromedial prefrontal cortex (Summerfield, Hassabis, & Maguire, 2009; Svoboda, McKinnon, & Levine, 2006). Dopamine activation is associated with a reduction in functional connectivity of the default mode network, involving the posterior cingulate gyrus (Conio et al., 2020).

Deficits in AM retrieval in schizophrenia can be revealed as a reduction in self-defining memories (SDMs) and an increase in over-general memory (OGM) (Allé et al., 2015; Berna et al., 2015; Nieto et al., 2019). Notably, the temporal distribution of AM shows difficulty retrieving vivid memories around illness onset (Elvevåg, Kerbs, Malley, Seeley, & Goldberg, 2003). OGM appears to be a transdiagnostic phenomenon that is observable in various disorders other than schizophrenia (Barry, Clark, & Maguire, 2021).

#### The role of involuntary AM in relapses

The retrieval of AM has been suggested to lie on a continuum between intentional and involuntary processes (Berntsen, 2010). Involuntary AM (IAM) retrieval constitutes a significant proportion of spontaneous cognition (Berntsen, 2023). IAM is considered an evolutionary earlier form of memory than AM and can arguably be observed in other primates (Allen & Fortin, 2013). Under the SAMP account, IAM retrieval is considered to be a key process involved in psychosis relapse and treatment resistance.

IAM retrieval involves external or internal cues triggering associative retrieval of past events based on the uniqueness of the cue memory, or ‘encoding-retrieval’, match (Berntsen, 2010, 2021, 2023). Commonly observed in everyday life, IAM involves the default mode network as in AM but there is, as expected, less activation of the prefrontal cortex (Hall et al., 2014; Hall, Gjedde, & Kupers, 2008). While IAM is mostly adaptive and functional, it can become dysfunctional when the content of the retrieved memory is distressing or disruptive (Berntsen, 2023). Patients with psychosis are more prone to experiencing IAM, particularly those triggered by internal rather than external cues (Allé, Berna, Danion, & Berntsen, 2020). Although IAM contents during remission are generally mundane, they are rated with higher self-relevance but lower ‘belief in actual occurrence’ (Allé et al., 2020). Not requiring intentional retrieval, the automatic involuntary characteristic makes IAM a prime candidate for mediating the reactivation of SAMP during psychosis relapse. So far, most AM/IAM studies have not specifically focused on SAMP. Future studies are required to distinguish between memories encoded during psychosis and nonpsychotic periods.

After consolidation, memory traces become relatively stable and are stored in the cortex independent of hippocampal involvement until they are reactivated (Goto, 2022). We propose that residual SAMP can be associatively reactivated as IAM. The probability of retrieval depends on the uniqueness of the encoding-retrieval match (distinctiveness of the cue-SAMP association, or SAMP potency) (Berntsen, 2023). The dynamics of memory retrieval have been described using associative network models (Rolls, 2021). One inherent feature of associative retrieval is that the retrieved memory can act as internal cues for further iterations of retrieval. In this way, an initial cue can trigger a cascade retrieval of related memories. Notably, this dynamics is consistent with the clinical observation of the rapid build-up of psychosis symptoms from their first appearance to full expression in a relapse (Emsley, Chiliza, Asmal, & Harvey, 2013). The threshold-crossing of such retrieval may depend on both the potency of SAMP and dopamine levels. In a normal dopaminergic state, higher potency in SAMP is required for retrieval. Meanwhile, in a high dopaminergic state, the congruent internal physiological and psychological context of the first psychotic episode is emulated, and retrieval of SAMP with lower potency may be facilitated.

During a relapse, new psychotic memories are added to existing SAMP to synthesize a more potent SAMP cluster. Subsequently, less dopamine elevation is required to trigger this extended SAMP cluster, which may account for the increasing antipsychotic resistance following relapses. From this perspective, refractory psychosis develops when the accrued SAMP becomes so extensive that it can be triggered by common everyday cues without dopamine elevation. In this state, psychosis can be driven by memory retrieval processes alone independent of dopamine activity and is therefore nonresponsive to dopamine-blocking medication. The resolution of psychotic symptoms would require nondopamine-related strategies such as normalization of the SAMP by extinction.

### Accommodation of SAMP in AM

Newly encoded memory interacts with existing memories in the brain in processes that have been described as assimilation and accommodation (Armelin, Heinemann, & de Hoz, 2017; McKenzie, Robinson, Herrera, Churchill, & Eichenbaum, 2013; Preston & Eichenbaum, 2013). New memories structurally similar to existing memory templates (schema) are assimilated as new exemplars in the existing schema (Takeuchi et al., 2022). A new memory that does not fit easily into the existing schema may initiate a revision of the schema to accommodate the new information (McKenzie et al., 2013). This requires an active process that involves the hippocampus and the detection of novelty reflected in the prediction error signal (Duszkiewicz et al., 2019; Goto, 2022; Kamiński et al., 2018; Shohamy & Adcock, 2010).

Recent high-resolution brain imaging studies have supported two convergent pathways in the hippocampus: (1) an entorhinal cortex-CA1 monosynaptic pathway and (2) a dentate-CA3-CA1 trisynaptic pathway (Lavenex & Amaral, 2000; Van Strien, Cappaert, & Witter, 2009). The entorhinal cortex-CA1 pathway encodes new memories while the dentate-CA3-CA1 pathway enables associative retrieval of old memory (Bakker, Kirwan, Miller, & Stark, 2008; Leutgeb & Leutgeb, 2007; Leutgeb, Leutgeb, Moser, & Moser, 2007; Yassa & Stark, 2011). CA1 may act as a mismatch comparator between the new memory and the retrieved memory (Duncan, Ketz, Inati, & Davachi, 2012). Further, the encoding and retrieval pathways may switch between competitive and integrative modes, as regulated by prediction error, as well as acetylcholine, dopamine, and noradrenergic activities (Richter, Chanales, & Kuhl, 2016; Schlichting, Mumford, & Preston, 2015; Schlichting & Preston, 2015). In the competitive mode, either encoding or retrieval is facilitated while the other is suppressed (Kesner & Rolls, 2015; Neunuebel & Knierim, 2014); in the integrative mode, cooperation between encoding and retrieval pathways facilitates interaction between new and old memories. These observations suggest that interactions between new and old memories in the brain are mediated through highly coordinated processes.

#### The roles of recovery style and nonbelieved memory

Clinical accounts after acute psychosis suggest that new event memories of psychosis are integrated with old nonpsychotic AM to different extents in people with different ‘recovery styles’ (Allé et al., 2015; McGlashan, 1987; Ridenour, Knauss, & Neal, 2021). In the ‘integrative’ recovery style, there is awareness of autobiographical continuity between the acute psychosis period and the nonpsychotic premorbid and remission periods. In contrast, in the ‘sealing over’ recovery style, patients ‘tend to isolate the psychotic experiences’ (McGlashan, 1987).

Consistent with the SAMP hypothesis, the integrative recovery style was associated with better long-term functional outcomes (McGlashan, 1987; Thompson, McGorry, & Harrigan, 2003). The success of integration can be reflected in the level of coherence of AM, which is found to be reduced in schizophrenia patients (Allé et al., 2015, 2016; Bisby, Horner, Bush, & Burgess, 2018). However, the integration between SAMP and nonpsychotic AM has rarely been specifically studied.

A relevant area of approach in understanding the possible processes in memory integration is the experience of ‘non-believed memory’, which has been explored mostly in nonclinical populations. Nonbelieved memories are vivid AMs of events once believed to be veridical but the belief of which is subsequently withdrawn (Scoboria et al., 2014; Scoboria, Nash, & Mazzoni, 2017). While previously thought to be rare and occur mostly in children (Otgaar, Wang, Fränken, & Howe, 2018), recent data suggest that nonbelieved memories are common in the general population (up to 35–50.6% in Li, Otgaar, Muris, & Chen, 2024). The withdrawal of a belief can occur as a result of social feedback; reappraisal of plausibility; attribution to a source other than memory; internal recollective characteristics; external details of the memory; general metacognitive belief about remembering; attributions about self or others, and personal motivation to alter belief (Scoboria et al., 2017). SAMP may undergo similar processes after remission.

Nonbelieved memories segregate into several subtypes based on how ‘non-believed’ and how ‘memory-like’ the representation is: (a) ‘high recollection with low belief’ characterizes a classical nonbelieved memory; (b) ‘high recollection and moderate belief’ characterizes a partial nonbelieved memory, which indicates that the belief is not completely relinquished by the weaker disconfirmatory evidence; (c) ‘moderate recollection and low belief profile’ characterizes a weaker recollection, which may be the result of strong disconfirmatory evidence (Scoboria et al., 2017).

Characterizing SAMP according to ‘recollection and belief’ profiling enables pragmatic subtyping of SAMP integration. These characterizations may mediate the relationship between SAMP and future relapse and treatment resistance. We hypothesize that a ‘high recollection-high belief’ (residual SAMP) would be associated with poorer outcomes (increased relapse propensity), with increasingly positive outcomes being indicated by ‘high recollection-moderate belief’ (partial integrated SAMP), ‘high recollection-low belief’ (well-integrated SAMP), and ‘low recollection-low belief’ (normalization of the well-integrated SAMP).

### The roles of salience and context exposure in the extinction of SAMP

Memory traces for episodic events are consolidated after a period in which hippocampus connectivity is required. Afterwards, the role of the hippocampus diminishes as the memory traces are transferred to the neocortex (Goto, 2022). Subsequent retrieval of the memory would require reactivation of the memory trace into a labile state with the possibility of reconsolidation (Lee, Nader, & Schiller, 2017). This reactivation process requires a state of salience involving a ‘prediction error’ (Lee et al., 2017). Through this process, newly acquired information reduces the tendency for the original response (including implicit associations and emotions). This normalizing effect is described as ‘extinction’ in animal studies across a wide range of vertebrate and invertebrate species.

Since psychosis and remission constitute different memory contexts, both psychologically and physiologically (Bouton, 2002), access to psychosis memory during remission may be compromised (Chen et al., 2023). Integration between SAMP and AM enables access to AM as a nonbelieved memory. Through memory integration, extinction is facilitated by access to the well-integrated SAMP-AM representations (extinction requires reactivation of the original memory trace) (Bouton, 2002; Lee et al., 2017). In contrast, poorly integrated SAMP is more likely to be left isolated and barred from extinction processes.

#### Normalization of SAMP requires normal salience and broad context exposure

Extinction has been observed in implicit and explicit memory in humans (Exton-McGuinness, Lee, & Reichelt, 2015; Lee, 2008; Lee et al., 2017). It is a highly context-dependent process (Bouton, 2000; Eisenhardt & Menzel, 2007) where a newly acquired normalized response is linked only to a specific context and may not be effective in other contexts. Therefore, the acquisition of ‘extinction’ learning across many different contexts may be important for efficacy (Papalini, Beckers, & Vervliet, 2020). Extinction requires new learning, which depends on the integrity of the dopamine-prediction error salience mechanisms and may be impeded by oversuppression of the dopamine e.g. by high-dose antipsychotic medication (Sumiyoshi, 2008). Antipsychotics at higher dosages (Malandain, Leygues, & Thibaut, 2022; McEvoy, Hogarty, & Steingard, 1991) may cause subjective dysphoria with an ‘inability to feel or think’ (Awad, 2019; Mizrahi et al., 2007) and disable the normal salience response. Clinical improvements in treatment-resistant patients transiting from high-dose first- and second-generation antipsychotics to clozapine may be partly accounted for by relief from dopamine suppression, enabling normalization processes to take place.

Based on the knowledge from extinction processes, normalization of SAMP could be facilitated by promoting exposure to normal life experiences, tackling social withdrawal, avoiding overmedication with antipsychotics, treatment of comorbid depression, and confronting ‘safety behaviour’ (as in cognitive behavioral therapy for psychosis).

### Clinical applications of SAMP in psychotic disorders and related conditions

The SAMP model has clinical implications for a spectrum of psychotic disorders, including but not limited to schizophrenia. Psychotic disorders manifest a substantial heterogeneity that is best handled by a multidimensional approach characterized by dimensions such as positive, negative, disorganization, affective and motor symptoms, each empirically associated with distinguishable brain substrates (Goghari, Sponheim, & MacDonald, 2010; Tandon, Nasrallah, & Keshavan, 2009). The SAMP perspective primarily addresses the positive symptom dimension.

**Figure 2.**
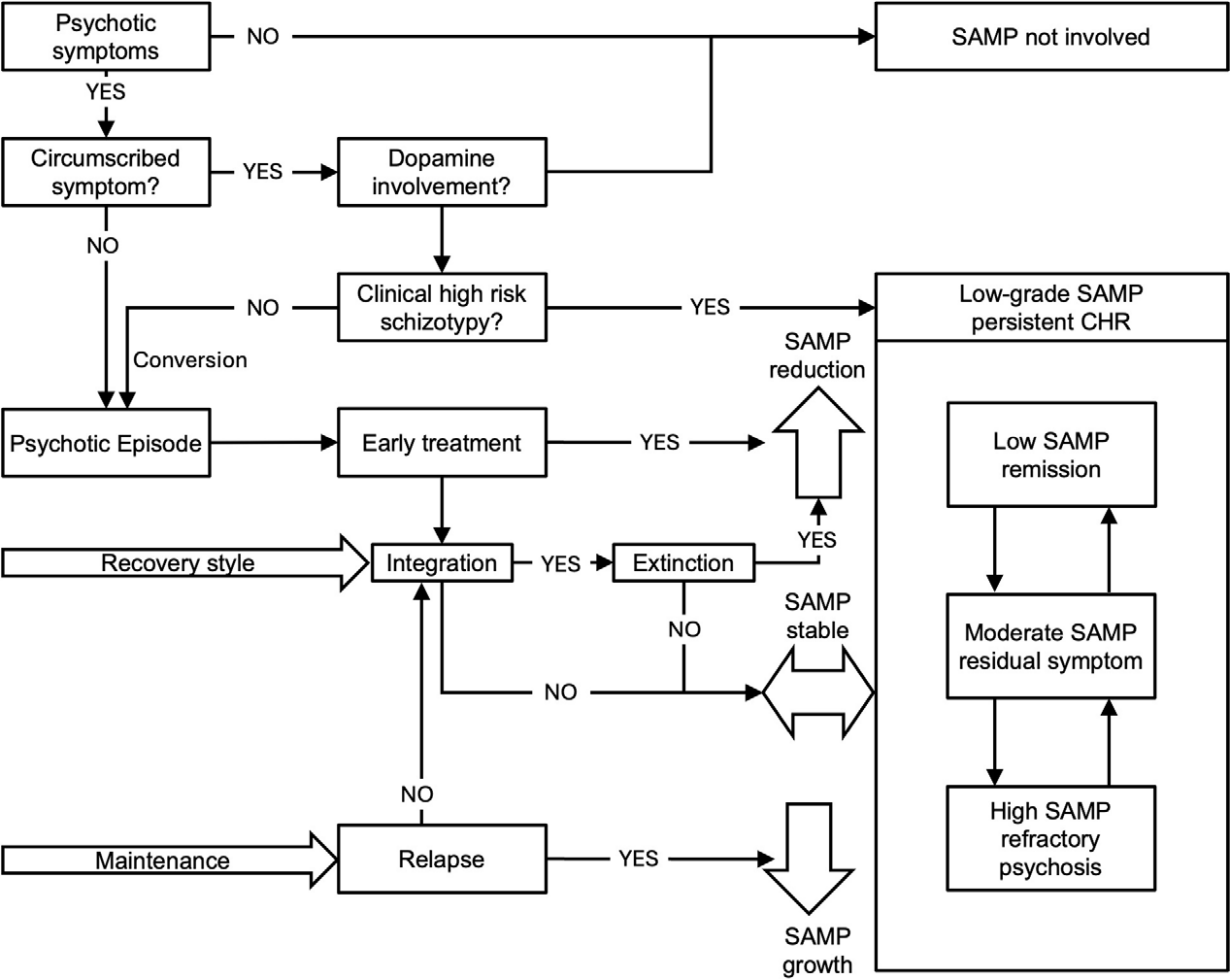
Flow diagram showing the relationship between clinical processes and the postulated SAMP status. Circumscribed psychotic symptoms not involving elevated dopamine activities do not lead to SAMP formation. Clinical high-risk states and schizotypal conditions may involve mild dopamine elevation leading to low-grade SAMP which may lead to persistent mild psychotic symptoms or decompensate into a frank psychotic episode. The first psychotic episode (FEP) results in SAMP formation. SAMP potency may depend on the content and duration of the FEP. Antipsychotic treatment results in remission except when SAMP potency is high, resulting in early treatment resistance. In remission, SAMP potency may reduce if integration with nonpsychotic autobiographical memory (AM) is good, and subsequent extinction is facilitated via exposure to normalized experiences. If SAMP-AM integration is poor, extinction is limited. If a relapse occurs, the new psychotic experience will strengthen the SAMP potency. Positive symptoms re-emergence depends on SAMP potency and dopamine state. A high-potency SAMP increases the risk of relapse and treatment resistance.

There is evidence that increased dopamine synthesis is involved in the first psychotic episodes (Cheng et al., 2020; Jauhar et al., 2017), during which SAMP formation is expected (Figure 2). The contents (cue-memory discriminability) and duration of active psychosis determine SAMP potency (see above; Berntsen, 2023). Upon remission, new SAMP is no longer actively formed. Nevertheless, as aforementioned, patients with different recovery styles (‘integrative’ or ‘sealing over’) attain different AM integrations between SAMP and lifetime memories (McGlashan, 1987; Thompson et al., 2003). Well-integrated SAMP is more accessible to the moderating effects of new normalized experiences (extinction), resulting in reduced SAMP potency, and thereby more favorable outcomes. Poor integration, overmedication, and negative symptoms compromise this process.

Furthermore, SAMP potency interacts with maintenance medication to determine relapse risks. With adequate maintenance, relapse can be minimized in most patients (Chen et al., 2010; Zipursky, Odejayi, Agid, & Remington, 2020) since context-dependent memory retrieval is inhibited by the changed dopamine psychophysiological context (Bouton, 2002). Although relapse takes place only in a small proportion of individuals with high SAMP potency (Rubio et al., 2020), we contend that the discontinuation of maintenance therapy would reinstate the dopamine salience psychophysiological context and lower the threshold for SAMP retrieval, leading to elevated risk of relapse even in patients with lower SAMP potency (Chen et al., 2010; Hui et al., 2018; Kishi et al., 2019). Notably, among those who remain on long-term maintenance medication and did not relapse, poor integration and extinction could also result in persistent SAMP, which might account for the lasting tendency for relapse even after long periods of remission (Chan et al., 2022; Tiihonen, Tanskanen, & Taipale, 2018). The role of memory in relapse is suggested by the similarity in psychosis themes and contents between relapse and previous episode(s) (Grunfeld et al., 2024). When a relapse occurs, new SAMP is appended onto the existing SAMP, resulting in an increase in SAMP potency and accounting for the observed increase in tendencies for further relapses and treatment refractoriness (Hui et al., 2018; Taipale et al., 2022).

The SAMP model is also relevant for those in a clinical high-risk (CHR) state. Some of them exhibit attenuated psychotic symptoms but do not cross the threshold into psychotic disorders. Individuals with CHR have been shown to demonstrate elevated dopamine synthesis capacities, particularly in those with higher symptom levels and those who eventually convert (Girgis et al., 2021; Howes et al., 2011, 2020). For those who did not convert into psychotic disorders, continuous low-grade psychotic experiences are expected to result in low-potency SAMP which integrates more easily with nonpsychotic AM. Consistent with the prediction from SAMP, there is a tendency for the persistence of attenuated psychotic symptoms with an increased risk of developing psychotic disorder (Addington et al., 2011; Woods et al., 2009). Similarly, the SAMP model predicts outcomes for schizotypal disorder, where long-standing attenuated psychotic symptoms are associated with evidence of dopamine dysfunction (Mohr & Ettinger, 2014) but less molecular imaging evidence of dopamine excess (Thompson et al., 2020). Indeed, the expected persistence of attenuated psychotic symptoms and increased risk of conversion to psychosis in schizotypy have also been reported (Addington et al., 2011; Albert et al., 2017; Woods et al., 2009).

Individuals in the nonclinical population may have isolated psychotic experiences (e.g., Morgellon disease, isolated hallucinations, as well as delusion-like ‘alien abduction’ and ‘past life’ experiences) without the typical decompensation seen in psychotic disorders (Clancy, McNally, Schacter, Lenzenweger, & Pitman, 2002; Meyersburg, Bogdan, Gallo, & McNally, 2009; Nunziato, Egeland, Gurman, & Henry, 2021). Studies have shown that dopamine elevation is not observed in people with isolated hallucinations (Howes et al., 2013). The lack of increased salience may explain why there is little cascading of the anomalous experience. Instead, these phenomena might be more related to metacognitive factors such as source memory weaknesses explained by a proneness to false memory (Clancy et al., 2002; Meyersburg et al., 2009) than to increased dopamine salience pathways.

### SAMP and PTSD symptoms

SAMP offers a parsimonious approach to understanding PTSD symptoms that are often also present in psychosis. Nevertheless, it should be highlighted that the phenomenological characteristics of psychotic and PTSD symptoms and the pathogenesis that underlies their emergence differ (Hardy, 2017; Samuelson, 2011). Higher-level SAMP retrieval extending to schema levels (delusions) is usually only found in psychotic disorders (OConghaile & DeLisi, 2015). Moreover, whereas psychotic hallucinations are predominantly verbal, more complex, and often linked with delusions, PTSD re-experiencing typically involves event memories with richer visual perceptual details (Bloomfield et al., 2021; Coughlan & Cannon, 2017; Morrison, Frame, & Larkin, 2003). How memories of traumatic events are encoded in the AM, the processes underlying the involuntary retrieval of these memories, and their roles in the reactivation of PTSD symptoms might differ from the processes observed in SAMP for psychotic disorders should be an area for further research. This would offer important information to advance the understanding of the similar and distinct pathways underlying psychosis and PTSD, as well as inform clinical innovations.

## Limitations and future directions

Given the lack of studies specifically addressing spurious AMs in psychosis, we opted for a narrative review of studies capturing the neurobiological and cognitive basis of psychotic disorders in the literature. The testable framework of SAMP offers directions for studying the long-term management of psychotic disorders in terms of minimization of residual SAMP by addressing minimizing the duration of active psychosis, facilitating integration between psychosis memory and nonpsychotic memory, and supporting normalization of psychosis memory through new learning across different life contexts and by avoiding overmedication.

While most of the work we reviewed is based on existing AM study methods in clinical and nonclinical populations, future studies should distinguish between AM encoded in the psychosis (SAMP) and other nonpsychotic life periods. A naturalistic longitudinal first episode study with AM measured at different time points may reveal further relationships between subtypes of SAMP (using profiling similar to that for nonbelieved memories described above) and clinical outcomes in terms of relapse, remission, recovery, and refractory psychosis. Brain imaging and electrophysiological measures targeting SAMP to extract individualized activity patterns in relevant brain areas during encoding, retrieval, and accommodation processes in AM tasks may add to phenomenological and cognitive observations. Importantly, focused investigations of AM integration and extinction processes (e.g., nonbelieved memory processes and context-dependent novel learning processes) via different methodological approaches can be critical for facilitating intervention development.

As explicated in this review, the SAMP approach could also be applied to a broader range of clinical conditions involving psychotic symptoms (e.g., substance-induced psychotic disorders and bipolar disorder with psychotic symptoms) in which dopamine-gated neuroplasticity could be involved. Notably, while the current approach focuses on AM, we consider AM to be integrated with implicit, semantic, and emotional memories. Spurious memory may also involve behavior, emotion, and semantic elements (e.g., Magioncalda et al., 2020). Concerning developmental trauma, content-specific symptoms have so far only been reported in a small proportion of cases (Bendall, Jackson, & Hulbert, 2010; Reiff, Castille, Muenzenmaier, & Link, 2012). Future studies are required to clarify its relationship with SAMP.

Further, current research suggests that dopamine encompasses multiple neurocognitive roles, including salience (Kutlu et al., 2021), prediction error (Millard, Bearden, Karlsgodt, & Sharpe, 2022), reward processing (Berridge, 2007), and neuroplasticity at the synaptic level (Speranza et al., 2021). Their potential integration has yet to be fully understood (Kutlu et al., 2021; Richter, Reinhard, Kraemer, & Gruber, 2020). In addition to classical ‘rewards’, it is recognized that dopamine also drives behavior through nonhedonic ‘incentives’ (Ventura, Morrone, & Puglisi-Allegra, 2007). Interestingly, the potential role of ‘salient information’ as a form of ‘incentive’ is compatible with the view that humans are ‘informavores’ driven by the consumption of information (Pylyshyn, 1984), a position aligned with the evolutionary social brain hypothesis (Dunbar, 2009). Consistent with this perspective, partial reinforcement paradigms showed that unpredictable rewards provide stronger motivational drivers for behavior (Harris, 2019). Integration between dopamine’s roles in handling ‘salient information’ and ‘rewards’ could be an important area for future exploration. The current SAMP model focused on the role of dopamine on neuroplasticity in psychosis. Future work can also explore the roles of other neurotransmitters (e.g., serotonin, noradrenaline, GABA, or acetylcholine) on SAMP.

## Conclusion

Memories of psychotic experiences are a relatively neglected area in psychosis research. We addressed the question of what happens to the memory traces encoded during psychotic episodes using new findings in the interaction between dopamine and the memory systems, as well as the emergent knowledge about the natural history of memory traces. We argue that this parsimonious account may be sufficient to address some key features in the longitudinal evolution of positive symptoms. The SAMP memory framework provides novel and pivotal conceptual tools that can facilitate the understanding of clinically relevant findings concerning the course of psychotic disorders, including the incremental accruement of dopamine-independent refractory psychotic symptoms after relapse, the need for active cross-contexts extinction in rehabilitation, and the relevance of the recovery style.
